# Employing the theory of planned behaviour to design an e-cigarette education resource for use in secondary schools

**DOI:** 10.1186/s12889-022-12674-3

**Published:** 2022-02-11

**Authors:** E. E. A. Simpson, J. Davison, J. Doherty, L. Dunwoody, C. McDowell, M. McLaughlin, S. Butter, M. Giles

**Affiliations:** 1 School of Psychology, Ulster University, Coleraine, Northern Ireland UK; 2 School of Nursing and Midwifery, Queens University of Belfast, Belfast, UK

**Keywords:** E-cigarettes; vaping, Theory of planned behaviour; young people, School-based intervention, Behavioural change taxonomy, Theoretical domains framework

## Abstract

**Background:**

An extended version of the theory of planned behaviour (TPB) was used to inform the design of a framework for an educational resource around e-cigarette use in young people.

**Methods:**

A sequential exploratory design was employed. In Phase 1, elicited behavioural, normative and control beliefs, via 7 focus groups with 51 participants, aged 11–16 years, identified salient beliefs around e-cigarette use. These were used to construct a questionnaire administered to 1511 young people aged 11–16 years, which determined predictors of e-cigarette use and ever use. In Phase 2, sociodemographic variables, e-cigarette knowledge, access, use, marketing and purchasing of e-cigarettes and smoking behaviour were also gathered. The composite findings from Phase 1 and 2 informed the design of a post primary educational resource in Phase 3 around e-cigarette use.

**Results:**

Current e-cigarette use was 4%, with almost 23% reporting ever use, suggesting current use is stable but experimentation may be increasing in this cohort. Sociodemographic variables, knowledge of e-cigarettes, smoking behaviour and TPB variables (direct and indirect measures of attitudes, subjective norm, and perceived behavioural control) accounted for 17% of the variance in current e-cigarette use, with higher intentions to use e-cigarettes within the next month, having the strongest impact on use (*p* < 0.001), followed by self-efficacy (*p =* 0.016). Sociodemographic and TPB variables accounted for 65% of the variance in intentions to use e-cigarettes in the next month; current e-cigarette use (*p* < 0.001), more positive attitudes (*p* < 0.001), stronger social influence (*p* < 0.001), higher self-efficacy (*p* < 0.001), higher control beliefs (*p* < 0.001) and greater motivation to use e-cigarettes (*p* < 0.001) were the main predictors of intentions. Phases 1 and 2 informed the mapping of key predictors of intentions and use of e-cigarettes onto the Theoretical Domains Framework, which identified appropriate intervention functions and behaviour change techniques.

**Conclusions:**

This paper is the first to bridge the theoretical-practice gap in an area of significant public health importance through the development of a framework for a novel theory driven school-based educational resource aimed at reducing experimentation and uptake of e-cigarette use in young people.

**Supplementary Information:**

The online version contains supplementary material available at 10.1186/s12889-022-12674-3.

## Introduction

The use of electronic cigarettes (e-cigarettes) has increased globally in young people [1, 2], at a time when traditional forms of smoking are declining [3, 4], and public health messages do not recommend their use in this group [5], hence such trends need to be closely monitored [1, 5, 6]. Although, regular use of e-cigarettes in young people remains low in the UK [7, 8] at around 4.9% in 2019, and mainly in smokers, experimentation is increasing and reported to be between 8.5 and 20% [7, 9, 10], compared to 16% experimentation with traditional cigarettes [3].

Adolescents appear to initiate e-cigarette use out of curiosity and their appealing attributes, in that they are “cool” and “less harmful”, compared to traditional cigarettes; whereas adults initiate their use mainly to quit smoking [11–14]. Therefore, there is no harm reduction application in e-cigarette use in young people [15, 16]. More worrying, current research is starting to link e-cigarette experimentation and use with more long-term nicotine addiction and subsequent initiation of smoking [10, 17–22], alcohol and drug use [10, 23, 24]. Some of the evidence is US based and may not be directly applicable to the UK due to different controls and regulations around e-cigarette production and control.

Since 2018, the Medicines and Healthcare products Regulatory Agency have set several controls around the sale and nicotine content of e-cigarettes. It is illegal to sell e-cigarettes to anyone under the age of 18 years in the UK, there are restrictions on advertising such products, and the maximum amount of nicotine allowed in an e-cigarette is 20 mg/ml. In comparison to the U.S. which does not have nicotine concentration restrictions and does not allow the sales of e-cigarettes to anyone under the age of 21 years. However, this has not prevented the illegal selling of super strength nicotine e-cigarettes to children in the UK, using social media to advertise such products, and raising concerns about the impact on health by Action on Smoking and Health (ASH, 2021) [25]. Adding to this concern, is the link between e-cigarettes and smoking which may be dose dependent, so the greater amount of nicotine in an e-cigarette the more likely it is to lead to traditional smoking in young people [19] and may be reinforced by the rewarding effects of vaping [26]. This has the potential to undo reduction trends in smoking behaviour in the UK and may have long term implications for health, therefore e-cigarette use in young people needs to be monitored.

The marketing of e-cigarettes may make them more appealing to young people, as they are presented on social media platforms via influencers [27, 28], and may be contributing to global increased use in this age group [29, 30]. In addition, the variety of devices and customised flavours designed to appeal to younger consumers (milkshake, chocolate, bubble-gum), promote experimentation with e-cigarettes in some studies [28, 31]. There are currently no restrictions on buying e-cigarettes with flavours in the UK, but other European countries are banning flavours, such as the Netherlands, in an attempt to reduce use in young people. More regular use of e-cigarettes in young people is also promoted by ease of purchase, absence of smoke, ease of concealment, parental smoking and lack of legislation controlling purchasing behaviour [13].

Safety concerns have emerged about the contents of some e-cigarettes, which typically include propylene glycol, vegetable glycerine, flavourings, and nicotine [32, 33]. There are over 7000 flavourings that can be added to e-cigarettes, the toxicity of which are unknown [15, 34], and some studies report additional harmful chemicals, such as formaldehyde and nickel [35]. It is not surprising that there is emerging evidence suggesting some e-cigarettes have the potential to impact negatively on respiratory, cardiovascular and brain function in adolescents, even with short term use [1, 29, 36]. Such findings make it an urgent public health requirement to better understand predictors of e-cigarette use and identify educational needs around e-cigarette use in schools, in an effort to promote informed choice and prevent uptake in young people [37].

Recommendations from a recent systematic review suggest that e-cigarette prevention and cessation programmes for young people need to be separate from tobacco campaigns and grounded in behaviour change theory [1, 38]. This need is met in the current study, as the theory of planned behaviour (TPB) [39, 40] was employed as the theoretical framework, with the sole focus on e-cigarette use. It is a leading behaviour change theory recognised by National Institute for Health and Care Excellence (NICE) for its effectiveness in the design and evaluation of behaviour change interventions [41, 42]. According to the theory, behaviour is determined by behavioural intentions, which in turn are influenced by three independent constructs, attitudes (advantages and disadvantages of engaging in a behaviour) subjective norms (SN - social influences on the behaviour) and perceived behavioural control (PBC - facilitators and barriers to a behaviour), often separated into measures of perceived control and self-efficacy) [39, 40]. A small number of recent studies have applied the TPB to e-cigarette use, but there are limitations as to what can be drawn from this work, because these studies are not full TPB applications, they have not followed the sequential framework recommended by Ajzen [39] and Francis et al. [40]. for the design and conduct of TPB studies [43]. The few that have used the TPB have done so with adult or student populations, with none of this work conducted amongst adolescents [43–45]. Also, many of these studies have only used single items to assess e-cigarette use, i.e. ‘Have you ever tried an e-cigarette?’ [7], or have been embedded within larger well-being surveys [46]. Our study addressed these limitations and gaps, and enabled identification of targets for intervention development through the design of a theory driven framework for an educational resource to be used in post primary schools. This hascombined theory and practice in an area of significant public health importance in the UK.

### Rationale for current study

The overriding aim of this research project was to assess prevalence and predictors of e-cigarette use and experimentation in young people aged 11–16 years in Northern Ireland (NI) Objectives: (1) An elicitation study was conducted to identify factors that influenced young peoples’ motivations to use e-cigarettes; (2) a TPB-based questionnaire was constructed from the elicited beliefs; (3) the questionnaire was administered to a representative sample of young people, which determined knowledge, attitudes, prevalence of e-cigarettes use, experimentation, and identified the potential predictors of use (4), this informed the design of an framework for an educational resource to be used in post primary schools, to prevent e-cigarette uptake and experimentation [37, 47]. This will be the first theory driven, evidenced based e-cigarette intervention for young people in the UK, designed to be incorporated into the curriculum [10].

## Materials and methods

Consistent with the advice of Ajzen [39] and Francis et al. [40], a sequential mixed methods design identified the key underlying beliefs around e-cigarette use, which was in keeping with other successful TPB school-based interventions that targeted adolescents [48]. This research was conducted in three Phases. Phase 1: An elicitation study, identified the indirect salient beliefs around e-cigarette use and involved a series of focus groups; Phase 2: A TPB questionnaire survey was based on the most common behavioural beliefs, social influences and control beliefs from Phase 1; Phase 3: Intervention Mapping informed the design of a framework for an educational resource aimed at enhancing young people’s intentions not to use e-cigarettes. This was achieved by targeting the major predictors of intentions and use of e-cigarettes, along with the theoretical domains framework and the Behaviour Change Techniques Taxonomy (BCTT), to guide the intervention functions and behaviour change techniques [49, 50].

### Phase 1: elicitation study

#### Aim

The aim of this Phase was to explore the salient beliefs that surrounded e-cigarette use in young people, it used qualitative methods and informed the design of a questionnaire based on the most frequently reported beliefs.

#### Design

Since young people do not form attitudes towards e-cigarettes in isolation [2], focus group methodology was deemed the most appropriate method to capture the dominant beliefs within this cohort. This allowed the researcher to experience the dynamics of the group as well as the practicalities and the sensitivities surrounding the target behaviour [51]. Further, such qualitative work enhanced the validity of questionnaires developed from their findings [52], which informed the basis of Phase 2 of this research.

#### Participants and procedure

Following ethical approval from the Ulster University Research Ethics Committee (UREC), data were collected during December 2016. A sample of post primary schools from each of the five Education Authority (EA) areas in NI was sought, and included children from different socio-economic groups, as well as schools in receipt of free school meals (FSM) (a social indicator of deprivation; Education Authority for NI, 2009 [53]. Contact was initially made with five post primary schools, and invitation letters that detailed the study aims, objectives and procedures, were emailed to each school, two schools agreeed to participate. The schools were asked to randomly select one class from each year group (years 8 to 12), and distributed parent and pupil information sheets and consent/assent forms, to all pupils in the selected class. Only those children who had completed parental consent and pupil assent took part in the focus group.

This resulted in seven focus groups, conducted by an experienced facilitator, involving a total of 51 young people aged 11–16 years (mean age = 13.45 years (SD = 1.346)); 22 were male and 29 females. The size of focus groups ranged from 3 to 10 participants, with an average of 7 participants in each group. In keeping with the format specified by Francis et al. [40] and Fishbein & Aizen, [39], nine open-ended questions were asked. The behaviour of interest (intentions to use e-cigarettes) needed to be clearly defined and specified, this was achieved in accordance with the TPBs TACT principles (Target: 11–16 year olds; Action: e-cigarette Use; Context: Everyday life; and Time: within the next month [39, 40]. The focus group questions tapped into the main constructs within the theory, and elicited accessible beliefs about attitudes (What do you believe are the advantages of using e-cigarettes?), SN (Are there any people or groups who would approve of you using an e-cigarette?) and PBC (What factors or circumstances would enable you to use e-cigarettes?) factors, questions are given in supplementary data 1.

Each focus group was conducted using strategies and recommendations by Gibson [54] and lasted approximately 30 to 40 min. At the start of each focus group, the facilitator introduced themselves, explained both the project and the format of the focus group, etiquette around taking turns to speak and welcomed any questions from participants. Participants were also invited to complete a socio-demographic questionnaire (age, gender, mother/father/guardian was in employment), and questions on smoking and e-cigarette use. The session then began with an introductory conversation about e-cigarettes, participants were asked if they had heard of e-cigarettes or seen them advertised. This helped to ensure that participants were relaxed and aided group dynamics, as it offered individuals the opportunity to speak and contribute to the discussions. All opinions were welcomed and respected. Data saturation was achieved (i.e. no new data or themes emerged), which suggested an adequate sample was reached [55]. All focus groups were audio recorded and transcribed verbatim.

#### Data analyses

Data were content analysed [56] and organised into emerging behavioural, normative and control beliefs in keeping with the TPB manual and Aizen’s recommendations [39, 40]. This was achieved by reading the transcripts several times, becoming familiar with their contents, coding the data, and identifying patterns in the data, which led to subthemes being established and labelled. This process was completed by two independent researchers who compared and discussed beliefs until a consensus was reached. The themes (Table 1) represented the “modal salient beliefs” of this cohort, also called the indirect beliefs about e-cigarettes, and were used to inform the construction of the TPB questionnaire. This involved a summative analysis of the themes to determine the order of frequency of beliefs within each construct [40], the most frequently occurring themes were included and used to formulate the belief-based (direct beliefs) measures of attitudes, SN and PBC that were used in the questionnaire, which required further investigation in a larger scale survey in Phase 2.. This purposeful approach to counting is recognised as good practice in qualitative research [57].

**Table 1:** Thematic analysis showing beliefs elicited and quotes from the focus group data about E-cigarette use in 11–16 year olds

Component	Theme	Example Quotation
**Attitude**	Health	*‘It’s better than smoking*. *. .there’s no like rocket fuel in them and all that, you know those other things that give you cancer and stuff. . .’* *‘They are still harmful; you are still smoking. You are inhaling something into your body so they are still affecting your lungs’.* *‘You probably get addicted to them with nicotine in it’.*
	Cessation	*‘I think they are good cause they stop you smoking’.* *‘It could take you off them (cigarettes) for a while. They are good cause they help you quit smoking’* *‘People use them to try to get off cigarettes.*
	Cost	*‘Price is really the worst thing like, I’ve just spent sixty quid on a new one yesterday’.* *‘Yeah, like they are all using e-cigarettes cause they can’t afford cigarettes now’.* *‘Some of them are really expensive…’*
	Appeal	*‘There’s no smell like when you smoke an e-cigarette like the smell doesn’t stick to you’.* *‘It’s just it’s fun. .. they are nice tasting. .. there are lots of nice flavours’.* *‘You can blow hoops like the wee kind of tricks you can do’.* *‘I just think they are class looking’.* *‘They are stylish to have’.*
	Safety	*‘You don’t know if it will explode when it’s charging. .. it could catch fire. .. some of them blow up in your face’.* *‘You don’t really know what’s in them. Some places might have their own house flavours.*. *you don’t know what’s in them’.* *‘They wouldn’t burn your house down like can happen with cigarettes’.*
**Subjective Norms**	Parents	*‘My mum would want me to use one’.* *‘Because they (parents) are trying to give you the best advice to keep yourself healthy’.* *‘they (parents) probably wouldn’t care if you’d an e-cigarette, but they care more if you’ve cigarettes’*
	Family	*‘Probably my uncle cause he, his children and stuff all do it’.*
	Friends	*‘Friends want you to do what they are doing…’.* *‘If your friends are using them.. .you are able to use theirs’.*
	Teachers	*‘Teachers cause they’re looking you to represent the school well and all that’.* *“I guess teachers, I don’t think they would like us to use them”.*
	Other professionals e.g. coaches	*“Probably like, the doctors, or like the dentists cause like your teeth are going to rot if you smoke”.* *“Yeah, ….. doctors”.*
**Perceived Behavioural Control**	Peer Pressure	*‘it’s a social thing, if everyone else is doing it as well.. ‘* *‘Kind of just want to fit in’.* *‘The ides of fitting in, like peer pressure..’*
	Perception	*‘Some people think they’re stylish to use’.* *‘I just think they are class looking’.* *‘Yeah it looks pretty cool’.* *‘…yeah cause you can get ones in really nice colours and stuff…’*
	Conformity	*‘If you are caught with it by your mum or dad or whatever its less serious than being caught with cigarettes’.* *‘If your parents allowed you to have one…’* *‘The law would prevent us using them.. .if you are our age you’re not allowed to use one.. .the government don’t allow us to use them’.* *‘We aren’t allowed to use them…it’s the law, we can’t buy them…’*
	Availability	*‘If your parents have one then you can use theirs’.* *‘Getting one as we aren’t allowed to buy them’.* *‘If your friends are using them. .. you are able to use theirs’.*
	Convenience	*‘You can just whip it out and smoke it anywhere’.* *‘It kind of saves you having to carry around cigarettes and lighters’.* *‘Easy to carry around, you can’t carry a cigarette around as it would burn a hole in your pocket.*
	Curiosity	*‘Just wanting to do it. Wanting to try it. .. to try something new’.* *‘Just something to fill your time’.* *‘Just to try it…’*

*N* = 51

## Results

All TPB constructs were reported to be relevant to e-cigarette use, see Table 1 for an overview of themes and sample quotes. Participants held attitudes about the advantages and disadvantages of using e-cigarettes. Contradictory views were discussed in relation to the health impact and potential as a quitting device. Of significance was the appeal of e-cigarettes amongst this cohort, as they were viewed as ‘cool’ and ‘stylish to use’. The ease with which e-cigarettes were obtained and the variety of flavours were also considered factors that encouraged experimentation and use. Participants were aware that it was illegal for them to purchase or use e-cigarettes, but their accessibility and provision from friends and parents greatly encouraged usage. When asked about the importance of significant others in relation to e-cigarette use, specific reference was made to friends and family, parents who were viewed as both approving and disapproving of their use. Participants reported they were less likely to get into trouble with parents if caught with an e-cigarette, as their parents perceived them to be less harmful in comparison to traditional cigarettes. The strong social influence from friends encouraged and facilitated e-cigarette use in social contexts, by borrowing friends’ devices or coming under peer pressure to use them. E-cigarettes were also regarded as being more convenient to use given that there were fewer restrictions placed on where they could be used, in addition to them being easy to carry in their pockets. The expense of purchasing the vaping device, as well as safety concerns surrounding the actual device (exploding batteries) were however viewed as factors that discouraged their use.

### Phase 2: questionnaire survey

#### Aim

The aim of this Phase was to continue the formative components of the research and developed a TPB questionnaire, based on the findings of Phase 1. This allowed the identification of the most significant predictors surrounding e-cigarette use and experimentation in young people aged 11–16 years, it informed which beliefs should be targeted to change intentions and behaviour and informed the design of an educational resource described in Phase 3 [58]. This was achieved by administering the TPB based questionnaire formulated from the salient beliefs elicited from Phase 1 of the study (Table 2).

**Table 2:** Salient beliefs elicited about e-cigarette use

**Attitudinal Beliefs**	
• A healthier alternative to smoking cigarettes	
• A cheaper alternative to smoking cigarettes	
• Less addictive than smoking cigarettes	
• A fun thing to do	
• A safer alternative to smoking cigarettes	
• Helping to quit smoking	
• Preventing the smell of smoke	
**Normative Beliefs**	
• My friends	
• My parents	
• Other members of my family	
• My teachers	
• My coaches	
**Control Beliefs**	
• Being in the company of my friends who own an e-cigarette	
• Curiosity of trying an e-cigarette	
• Providing me with the opportunity of looking cool	
• Having friends who use an e-cigarette	
• Having my parents’ permission to use an e-cigarette	
• Knowing they are against the law would prevent use	
• Not having enough money to buy an e-cigarette	
• Adhering to the school rules regarding e-cigarette use	
• Not owning an e-cigarette	
• Knowledge around long term impact on health	
• Getting into trouble with my parents	

#### Design

In keeping with TPB protocols, the TPB questionnaire included direct measures of attitudes, SN, PBC and intentions and behaviour [40]. Also, the indirect measures included behavioural beliefs and outcome evaluations, normative beliefs, and motivation to comply and control beliefs and perceived power to influence behaviour, all based on the salient beliefs’ items identified in Phase 1 for attitude, SN and PBC. In addition to TPB constructs, other variables relevant to the target behaviour were also included in the questionnaire (Table 3).

**Table 3:** TPB questionnaire items and alpha reliabilities

Construct	Number of items	Sample items
Intention	3	I intend (want to/going to) use an e-cigarette within the next month; (5) strongly agree (1) strongly disagree (Cronbach’s α = 0.97).
Attitude	4	Direct attitude: For me, using an e-cigarette within the next month would be very healthy (5)/very unhealthy (1); very good (5)/very bad (1); very foolish (1)/ very wise (5). (Cronbach’s α = 0.89).
	8	Indirect ‘belief-based’ attitude: Seven outcome evaluations (See Table 2), e.g. ‘safer alternative to cigarettes’: very good (+ 2) to very bad (− 2) multiplied by the corresponding behavioural belief, e.g. ‘using an e-cigarette would be a safer alternative to cigarettes’: very true (5) to very untrue (1).
Subjective Norm	4	Direct subjective norm: Most people who are important to me think that I should (would approve/would want me to) use an e-cigarette: ‘strongly agree’ (5) to strongly disagree (1). (Cronbach’s α = 0.67).
	6	Indirect subjective norm: Six normative beliefs (See Table 2) e.g. ‘my parents think that I should use an e-cigarette’: strongly agree (+ 2) to strongly disagree (− 2) multiplied by the corresponding motivation to comply e.g. ‘generally speaking I want to do what my parents want me to do’: strongly agree (5) to strongly disagree (1).
Perceived Behavioural Control	3	Direct perceived behavioural control: Whether I use an e-cigarette or not is entirely up to me: strongly agree (5) to strongly disagree (1); It is mostly up to me whether I use an e-cigarette or not: strongly agree (5) to strongly disagree (1); It is my decision whether I use an e-cigarette or not: strongly agree (5) to strongly disagree (1). (Cronbach’s α = 0.82).
	11	Indirect perceived behavioural control: Eleven control beliefs (See Table 2) e.g. The curiosity of trying an e-cigarette would encourage me to use an e-cigarette: very true (5) to very untrue (1)/ Adhering to the law would prevent me from using an e-cigarette: very true (1) to very untrue (5).
Self-efficacy	3	Direct self-efficacy I am confident that I could/I am sure that I could/It is easy for me to use an e-cigarette: strongly agree (5) to strongly disagree (1). (Cronbach’s α = 0.89).
Knowledge of e-cigarettes	7	The legal age to purchase an e-cigarette is 18 years/ E-cigarettes do not contain nicotine/E-cigarettes do not produce tar and carbon monoxide: True/False
E-cigarettes use	16	Do you/your mother/your father/your care giver use/ever used e-cigarettes Yes/No How often do you/did you use e-cigarettes (Occasionally, but not every day/1–2 times per week/3–4 times per week/Only on days that I am/was at school/everyday/weekends only)
Advertising/Exposure to e-cigarettes	3	Where did you first hear about or see e-cigarettes? Where have you seen e-cigarettes advertised? Have you been taught about e-cigarettes in school?
Smoking	4	Do you/your mother/father/care giver smoke tobacco cigarette: Yes/No How often do you smoke: Everyday/1–2 times per week/3–4 times per week/only the days I am at school/weekends only

#### Participants and procedure

The survey was undertaken between January 2018–January 2019. All post primary schools in NI were informed of the study objectives, via email and invited to participate. Ten percent of schools (*N* = 21) took part, which is similar to previous TPB research [48], and a financial incentive (£250) was given to each participating school.

Participants aged 11–16 years, both male and female, smokers and non-smokers, users and non-users of e-cigarettes were recruited from one randomly selected class from each year group (years 8–12), within each school, and provided a sample of 5 classes from each school. Each child received a study pack (an invitation letter, Participant Information Sheet, Consent form) to take home for one parent to complete. Those children who had parental consent and gave their assent on the day of testing, attended a room within the school and completed the questionnaire. The researcher was available during completion to support students and answer any questions.

The final sample comprised 1511 pupils, aged 11–16 years, across 21 secondary schools in NI, with a mean age of 13.5 years (Table 4). Just over half were female and from grammar schools, with all year groups (8–12) represented. A representative sample of all school types was achieved (i.e. grammar schools, secondary and integrated schools), with a mixture of management type (*n* = 7 were voluntary Grammar schools; *n* = 9 were state maintained schools; *n* = 2 grant maintained schools and *n* = 3 were Council for Catholic Maintained Schools), single sex (*n =* 3) and co-educational (*n* = 18).

**Table 4:** Sociodemographic, school variables and EC use in young people aged 11–16 years

Variable	%/M(SD)
**Pupil information**	
Sex %:	
Males	38%
Females	59%
Other/ Prefer not to say	3%
Age in years:	
Mean (SD)	13.5 (1.5)
E-cigarette information %:	
E-cigarette users	4%
Ever use	22%
**Parent information**	
Parental/guardian use of e-cigarettes:	
Mother/female guardian	8%
Father/male guardian	8%
In employment:	
Mother	81%
Father	90%
**School information:**	
School type:	
Secondary	33%
Grammar	59%
Integrated	8%
School year:	
Year 8 (11-12 yrs)	20%
Year 9 (12-13 yrs)	26%
Year 10 (13-14 yrs)	18%
Year 11 (14-15 yrs)	18%
Year 12 (15–16 yrs)	18%

*N* 1511, *EC* Electronic cigarettes

#### Questionnaire

The questionnaire consisted of five sections: 1) background information (see variables in Table 4); 2) e-cigarette use (past use, current use, parental use, exposure to advertising and marketing of e-cigarettes); 3) the predictors of e-cigarette use, using direct and belief-based measures of the TPB; 4) knowledge of e-cigarette; 5) to determine smoking behaviour (never smokers, experimenters, and occasional or regular smokers) and cessation. The TPB questionnaire was designed and scored in keeping with the TPB manual [48]. Table 3 provides information on the number of items included in each subscale, with sample questions and Likert answer formats employed for both direct and indirect measures. Intentions were scored by calculating the mean of the item scores. The direct measures of attitudes, SN and PBC: negatively worded items were recoded so that higher scores reflected more positive attitudes, greater social influence, and greater control respectively, then a mean of the item scores was calculated for each sub-scale. Indirect measures were calculated as follows: each behavioural, subjective norm and control beliefs were scored 1to 5, each was multiplied by the relevant evaluation, compliance and power scale items (scored − 2 to + 2). The products were summed for subscale scores. The internal reliability of each direct scale was calculated using Cronbach’s alpha and are presented in Table 3. The questions in sections 1, 2, 4 and 5 of the questionnaires were informed by a multidisciplinary Research Advisory Group (RAG) and a User Advisory Group (UAG). The RAG comprised key stakeholders, academics, researchers, public health professionals and policy makers. Whereas the UAG was made up of pupils, teachers, and parents. Both groups were involved in informing all aspects of the study, recommended as being good practice in intervention design [58]. The questionnaire was piloted on a sample of school children who had participated in the elicitation study, this was to check face validity of the questions and ensured that there were no issues or problems with the items. No subsequent changes were made to the questionnaire, so the pilot sample were included in the survey, in keeping with the TPB manual [40].

### Statistical analyses

Data were analysed using SPSS version 25, checked for normality [59]. The internal reliably of the TPB scales were assessed by Cronbach’s alpha [60]. All internal reliabilities for the direct TPB measures were within an acceptable range: intentions (α = 0.97), attitudes (α = 0.89), SN (α = 0.67), PBC (α = 0.82), self-efficacy (α = 0.89) and were in line with previous research [61, 62] that recommended above .5 for alpha reliabilities of subscales [40, 63]. Pearson’s bivariate correlations were computed prior to conducting the regression analyses to ensure that variables were not highly correlated. To determine predictors of current e-cigarette use, a hierarchical logistic regression analysis was conducted with current e-cigarette use as the dependent variable. The predictors were entered in five steps, with each step controlling for the variables in the previous step. Step one included all sociodemographic and school variables (sex, school year and type (grammar/secondary), mother/ female guardian and fathers/ male guardian use of e-cigarettes. In step two, knowledge of e-cigarettes was added; in step three the direct TPB beliefs (direct measures of attitude, SN, self-efficacy and PBC), and intentions in step four, with indirect TPB beliefs of attitude, SN, control beliefs in step five. To determine predictors of intentions to use e-cigarettes in the next month a hierarchical linear regression analysis was carried out with intentions to use as the dependent variable. The predictors were entered in six steps. Step one included socio-demographic and school variables; father and mothers e-cigarette use were entered in step two; current pupil e-cigarette use in step 3; knowledge of e-cigarettes was added in step four; step five included the addition of direct measures of the TPB constructs, and the indirect TPB beliefs in the sixth step. Checks on multicollinearity were performed for all hierarchical regression analyses, using collinearity diagnostics, two values were given, tolerance and VIF (variance inflation factor), both of which were within the acceptable values of greater than 0.1 and less than 10 respectively. The contribution of variables in the final step of the regression were examined, significant beta values reported and the semi-partial correlation squared (SPC^2^), which examined the unique contribution of the individual predictor to the overall variance in the predicted variable [64].

## Results

### Prevalence of e-cigarette use and experimentation

Over one fifth of the total sample (22.8%) reported ever having used an e-cigarette; 4.2% were current users and claimed to use e-cigarettes daily or several times per week. Around 7% agreed or strongly agreed that they intended to try e-cigarettes within the next month. Around 75% of users began to experiment with e-cigarettes between the ages of 12–14 years and for a small minority (1.6%), initiation began at the age of 10. Current and past use of e-cigarettes was higher among males (5.4 and 21.1%) than females (2.8 and 16.6%), although the association between gender and e-cigarette use was not significant. Interestingly, current and past use was highest amongst those who chose to describe themselves as ‘other’ in response to gender (1.3%); 15% of this group reported to be current users and 35% had experimented with e-cigarettes at some time in the past. E-cigarette use was more common among secondary compared to grammar school pupils. The association here was significant (***χ***^**2**^ = 16.55, df = 2, *p* < 0.01), with the former accommodating 5.5 and 22.5% of current and past users respectively, compared to 3.4 and 15.8% in the grammar school system. There were no differences between current users in those schools with high and low deprivation indicators (4.2 and 4.5%), 25% of those attending schools with high level of FSM reported to be past users compared to 17.4% in schools with less than 30% of pupils in receipt of free school meals. The association here was significant (***χ***^**2**^ = 7.44, df = 2, *P* < 0.05).

### Tobacco smoking and e-cigarette use and experimentation

Of those sampled, 3.5% reported that they currently smoked cigarettes. Of these, over half (56.6%) were currently using e-cigarettes and 30.2% had experimented with e-cigarettes in the past, meaning that only 13% of smokers had never tried e-cigarettes. Non-smokers were less likely to use e-cigarettes with 2.2% reporting to be regular users and 18% declaring to have tried e-cigarettes at some time in the past. The association between smoking and e-cigarette use was significant, such that smokers were more likely to use e-cigarettes (***χ***^**2**^ = 12.85, df = 4, *p* < 0.05).

E-cigarette use amongst young people was also associated with the behaviour of their parents. Specifically, 10.2% of young people with a mother/female guardian who used e-cigarettes were current users compared to 3.7% of those whose mother/female guardian did not use e-cigarettes. Similarly, 29.7% of young people whose mother/female guardian were users had previously experimented with e-cigarettes compared to 17.6% whose mother/female guardian were not. These associations were significant (***χ***^**2**^ = 24.17, df = 2, *P* < 0.001). Whilst the percentage differences were not significant for those whose fathers/male guardians used e-cigarettes, young people with fathers/male guardians who used e-cigarettes were more likely to be current users (7.3% compared to 4.0%) or to have experimented with e-cigarettes in the past (30.1% compared to 17.4%).

### Knowledge of e-cigarettes

Most respondents believed that e-cigarettes were addictive (85.6%), that they contain nicotine (76.9%), that the legal age of purchase was 18 years (78.8%) and that they were cheaper than cigarettes (67.7%). Approximately two thirds (64.9%) believed that e-cigarettes were an effective smoking cessation tool. However, respondents were less sure as to the by-products produced by e-cigarettes, less than half endorsed the claim that e-cigarettes produced tar and carbon monoxide (48.2%). Only a third of individuals believed that e-cigarettes were less harmful than cigarettes containing tobacco (34.6%).

### Marketing and purchasing of e-cigarettes

Less than 1 % of the sample (0.7%) endorsed never having heard about or seen e-cigarettes. Approximately two-thirds of the sample reported having first heard about or seen e-cigarettes via strangers using them (68.1%). This was followed by over a third of the sample endorsing they had seen them in the media (41.1%), promotional posters (40.3%), the internet (37.6%), friends using them (34.5%) and social media (34.1%). Having first seen or heard about e-cigarettes through friends (24.3%) and seeing family members using them (26.9%) were reported by around a quarter of the sample. A much smaller proportion of individuals reported first seeing or hearing about e-cigarettes through being told about them by family members (14.0%), being told about them by health professionals (5.4%) and other sources (3.1%).

The vast majority reported getting the device from a friend (54.5%). Around a tenth reported getting them from a family member or from home (10.5%), while smaller proportions reported buying them from shops (3.8%) or online (1.7%). Most individuals reported getting the e-cigarette top-up liquid from a friend (31.2%) rather than at shops (5.8%), family/home (5.2%) or online (1.5%).

### Predicting intentions to use e-cigarettes

Prior to conducting the regression analyses, a series of Pearson’s bivariate correlations were calculated for users and non-users, the results of which are displayed in Table 5, these showed small to medium correlations in the expected direction for the TPB constructs. To further explore these relationships, a hierarchical linear regression analysis was carried out with intentions to use e-cigarettes in the next month, as the criterion variable. The predictor variables were entered in six steps, see Table 6 for an overview of the findings at each step. In step one of the model, socio-demographic and school variables were predictive of intentions to e-cigarettes use, accounting for just over 7% of the variance in the R^2^; a significant change in the R^2^ occurred with the addition of father and mothers’ e-cigarettes use in step two accounting for 9% of the variance; in step three the addition of current pupil e-cigarettes use, accounted for almost 30% of the variance, but no change in the R^2^ with the addition of knowledge of e-cigarettes in step four. Step five included direct measures of the TPB constructs and led to a further increase in the R^2^, accounting for 57% of the variance; with the addition of the indirect TPB beliefs in the final sixth step, the variance in intentions explained increased to 65%. The unique contribution of variables to intentions in the final model was assessed by the square of the semi-partial correlation (spc^2^). In terms of the unique contribution to variability in intentions in the final model, the only predictors were attitude (spc^2^ = 0.041), SN (spc^2^ = 0.012), self-efficacy (spc^2^ = 0.016), and control beliefs (spc^2^ = 0.142), motivation to comply (spc^2^ = 0.029) and current e-cigarettes use (spc^2^ = 0.11). These results indicated that higher intention to use e-cigarettes in the next month was predicted by current e-cigarette use (β = 1.114, *p* < 0.001), having more positive attitudes to their use (β = 0.215, *p* < 0.001), being influenced by others (β = 0.147, *p* < 0.001), having greater self-efficacy (β = 0.094, *p* < 0.001) and control beliefs about e-cigarette use (β = 0.040, *p* < 0.001) and more motivated to use e-cigarettes (β = 0.008, *p* < 0.001).

**Table 5:** The relationships between TPB variables and knowledge of e-cigarettes in current users and non-users, aged 11–16 years

	Intentions	Attitude	Subjective Norm	Self-efficacy	PBC	Knowledge of EC
**Current users of EC**						
Intentions	1	**0.550**^**a**^	0.180	**0.418**^**a**^	0.128	0.036
Attitude		1	0.212	**0.323**^b^	0.175	0.067
Subjective Norm			1	−0.039	−0.099	0.082
Self-efficacy				1	**0.470**^a^	0.198
PBC					1	0.108
Knowledge of EC						1
**Non EC users**						
Intention	1	**0.577**^a^	**0.514**^a^	**0.487**^a^	**0.191**^a^	**0.079**^a^
Attitude		1	**0.483**^a^	**0.481**^a^	**0.208**^a^	**0.150**^a^
Subjective Norm			1	**0.382**^a^	**0.113**^a^	**0.096**^a^
Self-efficacy				1	**0.487**^a^	**0.092**^a^
PBC					1	0.040
Knowledge of EC						1

*N* 64, ^a^Correlation is significant at the 0.01 level (2-tailed). ^b^Correlation is significant at the 0.05 level (2-tailed). *EC* Electronic cigarettes, *PBC* Perceived behavioural control

**Table 6:** Summary of hierarchical linear regression analyses with socio-demographic, school variables, pupil, parent/guardian e-cigarette use, knowledge, direct and indirect TPB variables as predictors of intentions to use e-cigarettes in young people aged 11–16 years

Step	Predictor variables	***R***^***2***^	***∆R***^***2***^	***F, df and p***
1	Socio-demographic variables – sex, school year and type	0.076	0.076	**F**^**(8, 1386)**^ **= 14.22, < 0.001**
2	Mothers/female guardian and fathers male guardian EC use	0.097	0.021	**F**^**(2, 1384)**^ **= 16.41, < 0.001**
3	Current pupil EC use	0.295	0.197	**F**^**(1, 1383)**^ **= 386.7, < 0.001**
4	Knowledge of EC	0.296	0.001	F^(1, 1382)^ = 2.76, 0.097
5	Direct TPB measures	0.571	0.275	**F**^**(4, 1378)**^ **= 220.7, < 0.001**
6	Indirect TPB measures	0.653	0.083	**F**^**(3, 1375)**^ **= 109.2, < 0.001**

Step one: socio-demographic and school variables (sex, school year and type). Step two included the addition of mother/female guardian and fathers/male guardian e-cigarette use. Step three included pupil current e-cigarette use, step four saw the addition of knowledge about e-cigarettes. Step five included the addition of the direct measures of attitude, subjective norm, self-efficacy and PBC. Step sex the indirect measures of attitude, subjective norm, and control were added After step one, each subsequent step included the variable(s) from the previous step. Significant (*p* < 0.05) increases in *R*^*2*^ indicated in bold

### Explaining intentions to use e-cigarettes

In order to acquire a better understanding of why some young people were more likely to use e-cigarettes than others, and to corroborate the findings from the elicitation study, separate linear regression analyses were conducted with intention as the criterion variable for each and the salient beliefs underlying attitude, SN and PBC as the predictor variables in separate regressions respectively. Attitudinal beliefs accounted for 37% of the variance in intentions (F^(8, 1438)^ = 106.4, *p* < 0.001), with e-cigarettes being perceived as being more fun to use (β = 0.138, *p* < 0.001: spc^2^ = 0.18), cheaper to buy and safer to use than tobacco cigarettes (β = 0.054, *p* < 0.001: spc^2^ = 0.033; β = 0.029, *p* < 0.001: spc^2^ = 0.009) and less likely to get young people into trouble with parents (β = 0.031, *p* < 0.001: spc^2^ = 0.030). Normative beliefs accounted for almost 37% of the variance in intentions (F^(6, 1456)^ = 140.8, *p* < 0.001), with friends encouraging the use of e-cigarettes (β = 0.090, *p* < 0.001: spc^2^ = 0.099), followed by family (β = 0.040, *p* = 0.04: spc^2^ = 0.005), parents (β = 0.037, *p* = 0.005: spc^2^ = 0.005) and medical professionals (β = 0.037, *p* < 0.001: spc^2^ = 0.011). Control beliefs accounted for just over 54% of the variance in intentions (F^(11, 1422^) = 148.6, *p* < 0.001), parental permission to use e-cigarettes would facilitate their use (β = 0.291, *p* < 0.001: spc^2^ = 0.112), preventing use as a result of getting into trouble with parents for using them (β = 0.176, *p* < 0.001: spc^2^ = 0.046), curiosity to try e-cigarettes would encourage use (β = 0.112, *p* < 0.001: spc^2^ = 0.018), owning one would encourage use (β = 0.083, *p* < 0.001: spc^2^ = 0.012), when with friends who are using e-cigarettes, this would facilitate use (β = 0.066, *p* = 0.011: spc^2^ = 0.004), perceived as being cool to use e-cigarettes would facilitate their use (β = 0.047, *p* = 0.054: spc^2^ = 0.002), and adhering to the law would prevent their use (β = 0.041, *p* = 0.021: spc^2^ = 0.003). These findings supported the salient beliefs identified through the elicitation study in Phase 1, as being important influences on e-cigarette use.

### Predicting current e-cigarette use

This study provided an opportunity to determine the role played by the TPB constructs in predicting current e-cigarette use, albeit retrospectively. To this end, a hierarchical logistic regression analysis was conducted with current e-cigarette use as the dependent variable, this referred to use of e-cigarettes daily or several times per week. The predictors were entered in five steps, with each step controlling for the variables in the previous step. Step one: sociodemographic and school variables (sex, school year and type (grammar/secondary)) accounted for 2% of the variance (***χ***^**2**^ = 29.97, df = 8, *p* < 0.001) in current e-cigarette use. The addition of mother/ female guardian and fathers / male guardian use of e-cigarettes in step two accounted for 3% of the variance (***χ***^**2**^ = 11.25, df = 2, *p* = 0.004), knowledge of e-cigarettes was added in step three and did not account for any variance in use (***χ***^**2**^ = 1.30, df = 1, *p* = 0.253). The direct TPB beliefs (direct measures of attitude, SN, self-efficacy and PBC) and intentions were added in step four, accounted for 16% of the variance (***χ***^**2**^ = 188.43, df = 5, *p* < 0.001) and indirect TPB beliefs of attitude, SN, control beliefs did not account for any further variance in e-cigarettes use in step five (***χ***^**2**^ = 4.48, df = 3, *p* = 0.214). In the final step of the model (Table 7), the main predictors of e-cigarette use were intentions (β = 1.61, *p* < 0.001) and self-efficacy (β = 0.91, *p* = 0.016), the odds ratios of 5 and 2.48 respectively, showed that as intentions and self-efficacy in e-cigarette use increased, young people were 5 and 2.5 times more likely to use them.

**Table 7:** The final step in the hierarchical logistic regression analysis of predictors of current e-cigarette use in young people aged 11–16 years

							95% C.I. for EXP(B)	
Variables	B	S.E.	Wald	df	Sig.	Exp(B)	Lower	Upper
Female	−.222	1.166	.036	1	.849	.801	.081	7.875
Male	−.181	1.165	.024	1	.877	.834	.085	8.187
Other	−2.259	1.647	1.881	1	.170	.104	.004	2.637
Year 8 (11–12 yrs)	−.245	1.019	.058	1	.810	.783	.106	5.767
Year 9 (12-13 yrs)	−.621	.588	1.113	1	.291	.538	.170	1.703
Year 10 (13–14 yrs)	1.247	.664	3.526	1	.060	3.480	.947	12.787
Year 11 (14–15 yrs)	−.248	.595	.174	1	.677	.780	.243	2.506
School type	.520	.475	1.195	1	.274	1.681	.662	4.270
Mother’s/female guardian EC use	.299	.596	.252	1	.616	1.348	.419	4.337
Fathers/male guardian EC use	−1.042	.573	3.310	1	.069	.353	.115	1.084
EC Knowledge	−.032	.199	.025	1	.873	.969	.656	1.431
SN	−.177	.404	.192	1	.661	.838	.380	1.849
Attitude	.767	.400	3.678	1	.055	2.153	.983	4.714
PBC	.009	.339	.001	1	.978	1.010	.520	1.961
Self-efficacy	.908	.378	5.784	1	**.016**	2.481	1.183	5.201
Intention	1.610	.288	31.22	1	**.000**	5.004	2.844	8.802
Indirect measure of attitude	.001	.015	.009	1	.925	1.001	.973	1.031
Indirect SN	.029	.016	3.167	1	.075	1.029	.997	1.062
Indirect PBC	−.049	.040	1.461	1	.227	.952	.880	1.031
Constant	−8.127	3.966	4.199	1	.040	.000		

*N* 1511, School type = Grammar / Secondary School, *EC* Electronic cigarettes, *PBC* Perceived behavioural control, *SN* Subjective norm

### Phase 3: designing a framework for an educational resource on e-cigarettes to be used in schools

Phase 1 and Phase 2 provided a comprehensive definition of e-cigarette use in young people and outlined the main beliefs that underpinned the cognitive processes involved in the behaviour and identified what needed to change in relation to e-cigarette use. For example, attitudes that e-cigarettes were safer than traditional cigarettes needed to be challenged as e-cigarettes contain nicotine, which is addictive and linked to future cigarette smoking in adolescents [29].

According to Fishbein and Aizen [65], the next step in employing the TPB, involved reviewing the main predictors of intentions or behaviour, identified in Phase 2, to inform targets for an intervention. While theories like the TPB are helpful in terms of identifying facilitators and barriers to e-cigarette use, they do not specify how to change such predictors, specifically to bring about a change in behaviour [65–67]. In order to guide the behavioural change process [66], the TPB was used with an integrative framework, the Theoretical Domains Framework (TDF) [49]. Hence the main constructs of the TPB, all of which were identified as predictors on intentions to use e-cigarettes, were mapped onto components of the TDF [49]. This identified intervention functions, levers that can optimise change, via empirically supported behaviour change techniques that map to each intervention function [66]. This process of mapping the components of change onto the intervention functions and suitable theory driven BCTs was thought to produce more effective intervention design and behaviour change [49, 68], and is currently lacking in e-cigarette interventions for use in young people focusing on prevention of uptake and cessation [38]. This has been described in more detail below.

### Aim

A primary aim of Phase 3 was to design a TPB theory driven framework for an educational resource to discourage uptake and experimentation with e-cigarettes in young people, whilst also facilitating cessation in those already using e-cigarettes.

### Design

Phase 3 focused on the identification of the intervention’s functions and suitable BCTs that were included in the educational resource. An overview of this process is given in Table 8.

**Table 8:** Mapping the TBP constructs that predict e-cigarette use and intentions onto the TDF, intervention functions and suitable BCTs for inclusion in the intervention

TPB constructs	Predictors of intentions for eachTBP constructs	TPB constructs mapped onto the TDF	Intervention function	BCTs to be used to address intervention functions	APPEASE criteria	Content examples
Intentions	To use e-cigarettes in the next month	Intentions	Education Persuasion Incentivisation Coercion Modelling	Information about the health consequences. Antecedents Information about social and environmental consequences of behaviour	Yes Yes No No Yes	Encourage young people to intend to abstain from e-cigarette use. Refusal rehearsal. Embed abstention from e-cigarette use within the School.
Attitudes	Fun Cheap Safe Less likely to get into trouble with parents	Beliefs about consequences Knowledge	Education Persuasion Modelling	Information about the health consequences. Antecedents Information about social and environmental consequences of behaviour	Yes Yes Yes	Provide information on: -health implications of e-cigarette use and non-use. - outline links to nicotine and addiction smoking. Use media to identify examples of people to imitate or aspire to that do not use e-cigarettes.
Subjective norms	Friends Family Parents Medics	Social influences	Restriction Environmental restructuring Modelling Enablement	Restructuring the physical environment Demonstration of behaviour Social support Problem solving Action planning	Yes Yes Yes Yes	Raise awareness of social contexts that might lead to opportunities for experimenting with e-cigarettes. Social support to prevent use. Encourage others to abstain Reduce social acceptability.Provide information to parents on risk of e-cigarette use in young people. Raising awareness of parental roles in preventing uptake. Raising awareness of the need to restrict access to e-cigarettes in the home. Monitoring behaviour.
PBC: • Self-efficacy	Facilitators: Parent permission Curiosity Owning one Friends owning one Being cool Barriers: Parent getting into trouble Illegal purchase	Beliefs about capabilities	Education Persuasion Modelling Enablement	Information about health consequences Antecedents Information about social and environmental consequences of behaviour Demonstration of behaviour Social support Problem solving Action planning Restructuring the physical environment	Yes Yes Yes Yes	Enhance refusal self-efficacy Impart skills required to say no to e-cigarette experimentation Suggest to parents to discourage their children from using e-cigarettes via an information leaflet on their role in health behaviours
• Control beliefs		Environmental context & resources	Training Restriction Environmental restructuring enablement	Restructuring the physical environment	No Yes Yes Yes	Provide information on legislation around the use of e-cigarettes in young people. Raise safety concerns about e-cigarettes bought online. Create the expectation that there is a cost or punishment associated with the use of e-cigarettes. Embed these components in a school-based policy around the use of e-cigarettes in school

APPEASE criteria include considerations around affordability, practicability, effectiveness and cost-effectiveness, acceptability, side-effects/ safety and equity

### Procedure

This section describes the mapping the main TPB predictors of intentions and e-cigarette use, identified in Phase 2, onto the TDF in order to identify the intervention functions. The main predictors of current e-cigarette use were intentions and self-efficacy; the main predictors of intentions to use e-cigarettes were attitudes, SN and self-efficacy and control beliefs, so these were mapped onto the TDF, (Table 8). These predictor variables mapped onto 6 of the 14 TDF components, intentions, beliefs about consequences, knowledge, social influences, beliefs about capabilities, along with environmental context and resources.

The next step in this process was to identify the pertinent intervention functions that corresponded to the 6 identified TDF components (Table 8), which were education, persuasion, incentivisation, coercion, modelling, restriction, environmental restructuring, enablement, and training. To decide the appropriateness of the intervention functions for use in the school context, the APPEASE criteria (affordability, practicability, effectiveness and cost-effectiveness, acceptability, side-effects/ safety, and equity) was applied to each of the intervention functions in turn, (the outcome can be found in Table 8), to enhance the appropriateness and suitability of the components of the intervention [69]. It was acknowledged that education needed to be included, to allow young people to make more informed decisions about e-cigarette use. Hence information was included about the risks and benefits of e-cigarette use in young people (attitudes), along with increased understanding of social contexts that might facilitate e-cigarette use (SN), and advice on how to avoid or say no to e-cigarettes (SE). Persuasion not to experiment with e-cigarettes was included to reduce intentions to use e-cigarettes (intentions), to hold less favourable attitudes about their use (attitudes) and prevent use in social contexts (SE). Modelling non-use and resistance to using e-cigarettes was included to facilitate discussions around use, to enhance informed choice, and to raise awareness of the social impact on behaviour and enhance greater controllability and self-efficacy around e-cigarettes use. Restriction around access to e-cigarettes was included in relation to the environment (accessibility and the law – control beliefs), and in social contexts, where significant others were promoting their use (friends and family – SN). The inclusion of environmental restructuring addressed making accessibility to e-cigarettes more difficult for young people, via identified barriers such as, the need for parental permission, along with a ban on purchasing e-cigarettes and their liquids. Enablement was employed to equip young people with the social skills, support, and knowledge to make more informed choices about e-cigarette use and to practice refusal techniques (SE). Incentivisation, coercion and training were considered inappropriate in this context and were not included in the intervention design.

The next step was to select the most appropriate BCTs for each of the intervention functions. Twelve of the theoretical domains within the TDF, and their corresponding intervention functions, have been linked to 59 BCTs from the Behaviour Change Techniques Taxonomy v 1 [67, 70] and the Theory Techniques Tool [71]. Previous research identified the most frequently used BCTs to target each intervention function, and empirical evidence to support their effectiveness with regards mechanisms of action [72, 73]. Utilising BCTs provided a standardised description of the components for the framework to make replicable for future studies and has been recognised as good practice in intervention design [70]. Table 8 provides an overview of the BCTs chosen to address the intervention functions and a description of what the framework entails. The content and included BCTs are similar to a recent American intervention that was successful in reducing e-cigarette uptake in middle school pupils, but employed the social cognition model [74, 75].

The framework for the educational resource will facilitate co-production of materials with all stakeholders and will consist of a combination of online resources and activities for use in the classroom. For it to be employed successfully and be impactful, it will include a multi-modal approach to target young people, their parents, and the school context, such an approach has been employed successfully in previous school-based e-cigarette prevention campaigns in the US [74]. Teachers will need training on how to use the materials correctly and it will include lesson plans, specific sources of information, and group activities for each session. The sessions will target the TPB predictors of behaviour and intentions and should be easily incorporated into the curriculum.

Three lessons for pupils: Firstly, focusing on attitudes, this will include information about the health risks and benefits associated with e-cigarette use, the ingredients in e-cigarettes, the risks of nicotine addiction and gateway to future smoking of traditional cigarettes. Potentially aiming to decrease the positive attitudes surround e-cigarette use, as this has been found to promote initiation of use [76]. Discussion and activities to promote informed choice. Secondly, social influences will be considered through discussion activities considering social situations that might promote experimentation with e-cigarettes. How to manage peer pressure and role play to practice refusal and creating a social context where e-cigarette use is not socially acceptable, this is essential, as social networks of support that promote none-use of e-cigarettes have been found to reduce e-cigarette use in a recent intervention to prevent uptake [74]. Also raising awareness in parents that they have the potential to promote or restrict e-cigarette use in their children. Thirdly, behavioural control will involve discussions around e-cigarette use and the need to abstain from use in young people, promoting a sense of individual control and confidence to refuse e-cigarettes when offered them. This will promote resilience and self-efficacy around abstaining from using e-cigarettes, through discussion and practicing refusal. In addition, raising awareness of the safety and legislation around selling e-cigarettes to young people is important, as it was identified as a barrier to use. Lessons in the classroom focusing on education around e-cigarette use have been successful in changing attitudes, raising the risk of nicotine addiction and reduced intentions to use e-cigarettes [38, 74].

Parents are often regarded as active agents of change for the child’s behaviour and health [77], for this reason parents need to be educated and informed of the potential part they can play in their child’s use and experimentation with e-cigarettes. A health information leaflet will be developed for parents to raise awareness and understanding of the following: the health implications of e-cigarette use in young people; the impact on parental use of e-cigarettes as a facilitator to use in their children; and lastly information on how parents may prevent the uptake of e-cigarettes in their children by setting sanctions for use and creating an environment where it is not acceptable to use them. Parental involvement has been used previously in e-cigarette prevention [74].

Some work is required generally within the school context to help schools make changes to the school ethos and environment around e-cigarette use. To develop school policies and to promote environments where using e-cigarettes are not socially acceptable and come with sanctions. To create a pupil charter where abstaining from using e-cigarettes is agreed with pupils as a key goal. Embedding teaching around e-cigarettes into the curriculum and providing resources to facilitate and support this teaching. Providing visual aids and resources (posters) that are anti e-cigarette use in young people. Providing information and quit signposting for those who want to give up e-cigarettes.

## Discussion

Employing the TPB has provided a more comprehensive understanding of the psychosocial processes involved in e-cigarette use, and intentions to use e-cigarettes, in young people. To our knowledge, this is one of the first TPB studies to use an exploratory sequential design to inform the development of a framework for an educational resource focusing solely on e-cigarettes for use in post primary schools in the UK. This research was conducted in three phrases, Phase 1 and 2 clearly defined e-cigarette use in young people in Northern Ireland and identified underlying factors that mediated current e-cigarette use (intentions and self-efficacy) and intentions to use e-cigarettes (attitudes, SN, self-efficacy, and control beliefs) in this cohort. From the combined results of Phase 1 and Phase 2, which adhered strictly to the recommendations for using the TPB framework [39, 40], and in conjunction with the TDF [49], six key intervention functions (education, persuasion, modelling, restriction, environmental restructuring, enablement) and eight appropriate BCTs were identified (information about the health consequences, antecedents to e-cigarette use, information about social and environmental consequences of e-cigarette use, restructuring the physical environment, demonstration of behaviour, social support, problem solving, action planning), for inclusion in the framework for an educational resource in Phase 3. A recent review suggested that e-cigarette prevention and cessation programmes for young people needs to be theory driven, and separate from tobacco campaigns [38], this framework has addressed that gap in the literature.

Phase 1 & 2 of this research provided an overview of the extent of current use and experimentation with e-cigarettes in young people in Northern Ireland. Four percent of the sample reported to be regular e-cigarette users, which is comparable to prevalence rates of 4.9% reported among young people in Great Britain [3] and a slight increase from a separate survey in Northern Ireland of 3% [78]. In this sample experimentation with e-cigarettes was higher at 22%, compared to recent findings of between 8.5 and 15% reported in Great Britain [79, 80] and may be in line with recent research, which indicates that e-cigarette experimentation across the UK and US has overtaken traditional smoking [81]. In the current study, e-cigarette experimentation,, was almost double that reported for tobacco smoking in this age group [7, 46, 78], and is a worrying statistic. In addition, over 20% of non-smokers reported that they were current or past users of e-cigarettes, and supported claims that a notable proportion of adolescents who have never smoked have tried an e-cigarette [82]. With the e-cigarette market ever evolving and many devices and liquids developed to be appealing to young people [28], there is a need for longitudinal studies in Northern Ireland to monitor use, due to the risk of nicotine addiction and the possibility of it leading to subsequent smoking of traditional cigarettes [10, 18, 20].

Phase 2 also provided insights into the personal, social and control beliefs underlying intentions to use e-cigarettes. The TPB has a proven track record for exploring and measuring adolescent smoking behaviour [83–86], thus adding validity to our theoretical approach applied to e-cigarettes. Whilst a small number of recent studies have applied the TPB to e-cigarette use, there are limitations as to what can be drawn from that work, as they have not followed the recommended steps as identified by Ajzen [39] and Francis [40] for the design and conduct of TPB studies [43]. The few that have used the TPB have done so with adult or student populations, with paucity of work conducted amongst adolescents [43–45]. The current research addressed gaps in the literature, and also enabled identification of targets for intervention development, another major strength to this research and application of the TPB.

Holding more positive attitudes about e-cigarettes has recently been found to predict their use in adolescents [76]. The current study lends further support to these findings as the main attitudinal predictors of intentions to use e-cigarettes were more positive in nature, such as e-cigarettes being regarded as more fun to use, cheaper and safer than traditional cigarettes and less likely to get the young people into trouble with their parents, if caught using them. Previous studies have found similar results. Specifically, e-cigarettes have been reported to be a safer and healthier alternative to cigarettes [87, 88], less addictive [89, 90], cheaper and more convenient to use [91, 92]. Whilst there was some awareness of the debate around the health and safety implications in the qualitative aspects of the current study, with some participants describing them as harmful, it was the positive attitudes that predicted intentions to use e-cigarettes, which is in keeping with previous research [76]. As such, education around e-cigarette use is needed to present a more balanced view of e-cigarettes and address potential risks as well as benefits to enhance informed choices around their use.

This seems to be particularly important given that evidence is accumulating to suggest that the use of nicotine-based e-cigarettes in this age group may lead to a nicotine addiction and subsequent uptake of smoking traditional cigarettes [10, 17, 18, 93]. E-cigarettes have been linked to the normalisation and greater acceptance of smoking type behaviours in young people, which could further contribute to the uptake of traditional forms of smoking [10]. In some countries, e-cigarette use in young people has been linked to alcohol and drug use [10, 23, 24]. Although, some of the evidence is US based, and not directly applicable to NI, it does highlight potential issues and the need for monitoring to ensure it does not happen. This coupled with emerging evidence suggesting they have the potential to impact on respiratory, cardiovascular and brain function in adolescents, with short term use [1, 29, 36], makes it more important to address the reason why young people use e-cigarettes in an educational context [38].

In the current study, there was a strong normative influence on young people to use e-cigarettes, especially from friends and family, which also supports pervious work in this area [94]. This is in keeping with the strong peer influence on adolescent smoking, which is well established [95]. This played out in several significant ways. Specifically, the influence of one’s friends was the most significant normative predictor of intentions to use e-cigarettes and the encouragement provided by friends was also identified as a significant facilitator. The influence of peer pressure was also apparent in the qualitative data with the young people acknowledging e-cigarette use as a social behaviour subjected by peer-pressure, and the need to ‘fit in’ [91, 96]. Having parental permission significantly facilitated the use of e-cigarettes in this sample, whilst ‘the fear of getting into trouble with one’s parents’ and ‘adhering to the law’ were significant inhibitors, similar to previous research [97]. This together with the finding that users were more likely to have parents who used e-cigarettes and who gave their permission to use their devices suggests that parental attitudes and vaping behaviour is significantly associated with e-cigarette use in young people [98, 99]. Further, parental e-cigarette use, and tobacco smoking was associated with e-cigarette use in young people, as previous studies have found [100, 101]. Of course, this finding has policy implications; and public health authorities need to weigh up the balance between encouraging e-cigarette use by adult smokers and how this might affect uptake in young people.

The influence of control was also apparent in the role played by self-efficacy which was found to be a significant predictor of both current e-cigarette use and intentions to smoke e-cigarettes within the next month. Self-efficacy is important when targeting beliefs to change behaviour, as it is associated with feeling empowered and having control over one’s behaviour and the confidence in the choice that one makes and is vital for promoting resistance to potential harmful behaviours [102].

General control belief can be broken down into facilitators and barriers to e-cigarette use. In the current study, the main control beliefs that acted as facilitators were parental permission, curiosity, owning an e-cigarette, friends using e-cigarettes, perceived as being “cool”. Those control beliefs that acted as barriers were, getting into trouble with parents and breaking the law. In other words, having friends and parents who smoked e-cigarettes facilitated access, as it provided young people with opportunities to use their devices [72, 87, 103]. This in turn served to enhance their confidence in their ability to use them. Indeed, some people commented ‘if your friends are using them or your parents have one, you can use theirs’, see also [7, 104–106]. This appeared to be reinforced by the belief that their parents perceived e-cigarettes as a less harmful alternatives to smoking.

Considering the need for theory driven programmes that focus on e-cigarettes for young people [2, 38], this study identified several targets for intervention by mapping components of the TPB, that predict intentions to use e-cigarettes onto the TDF, to establish intervention functions and appropriate BCTs for inclusion in the framework for educational resource. Of primary importance is holding positive attitudes to e-cigarettes, the role played by one’s significant others, particularly one’s parents and friends, and the empowering of young people to abstain from using e-cigarettes and reducing accessibility of these products in this age group. A combination of approaches may be employed to achieve these objectives including the use of education, restriction, modelling and environmental restructuring, all used in previous tobacco prevention campaigns [107, 108] and in a recent e-cigarette prevention programme [75]. In the context of a framework for an educational resource, similar to the ASSIST intervention in the context of smoking cessation [109], the aim is to develop less favourable attitudes, make e-cigarettes more socially unacceptable for recreational use in young people and promote refusal skills, around the use of e-cigarettes. Such approaches have worked well in other health promotion programmes aimed at tobacco prevention with some information on e-cigarettes [38]. To move this educational resource forward, and in keeping with previous prevention campaigns for smoking and a recent one for e-cigarettes in the US [75], the logistics of the educational resource need to be agreed and co-produced with the RAG and UAG [110, 111], that were part of the current study. A multi-modal approach should be adopted to target schools, pupils and parents [38], to co-produce a resource that is fit for purpose and easily embedded within the curriculum.

### Strengths

The TPB is one of the most widely used theories of behaviour change and has been recognised by the NICE guidelines as good practice for intervention design [41, 42]. One of the theory’s main strengths, is its flexibility, as it allows for the inclusion of additional predictor variables such as sociodemographic characteristics, knowledge, and other behaviours, which is how it was used in the current study. The TPB has a proven track record for exploring and measuring adolescent smoking behaviour [83–86], so this adds further validity to this theoretical approach employed in the current study This study provides support for the application of the TPB framework to e-cigarette use in young people, as 64% of the variance in intentions to use e-cigarettes was explained by all of the TPB constructs, a finding which reiterates previous research [96, 112]. The importance of these constructs was also assessed in relation to current e-cigarette use (albeit retrospectively), addressing a criticism which is often levied at the TPB [107]. Using the TPB framework, in conjunction with the TDF and the BCTTvs1, enabled identification of appropriate BCTs to target key beliefs around e-cigarette use, in the design of a framework for an educational resource to be used in schools. The educational resource will hopefully help to achieve public health priorities in Northern Ireland. In this jurisdiction, the Public Health Agency recognise that e-cigarettes may have health benefits for smokers but do not regard e-cigarettes as safer than established smoking cessation tools and do not advocate for use in young people or as a smoking cessation tool.

### Limitations

The TPB is not without its critics, but no one theoretical framework can explain all variation in behaviour. Its focus on rational reasoning, excluding the influence of unconscious processes that may limit its ability to explain unplanned e-cigarettes use [113]. However, the amount of variance explained in the present context was comparable with previous research, which supports its use with young people [114]. Criticism of the theory has also centred on the use of TPB questionnaires, which are typically long and include the use of outcome expectancy measures, which are often viewed by respondents as somewhat repetitive [62]. This can negatively impact on responding. In order to combat this in the current study, participants’ behaviour, during completion was closely monitored; emphasis was given to the importance of honesty and personal opinion. As such, it was stressed that there were no right, or wrong answers and the response formats were varied across the questions to prevent response bias [39]. The sample focused on younger pupils (with an average age of 13 years), due to GCSE commitments in older pupils. Since the study findings suggests that e-cigarette use increased with age; future research should aim to ensure that older pupils are represented. Further, longitudinal evaluative research is needed to determine trajectories of e-cigarette use in adolescents, to establish links to nicotine addiction and links to traditional smoking, as well as co-production and establishing the effectiveness of the educational intervention to prevent uptake in this age group.

## Conclusion

This study has provided a unique opportunity to map the findings against the most well-respected behavioural change frameworks to inform the design of a framework for an educational intervention, which should be of use to key stakeholders in the areas of education and public health interested in reducing the prevalence of e-cigarette use in young people.

This framework addressed the key attitudes, social and control influences that promote e-cigarette use in this cohort, while presenting a balanced approach to facilitate harm reduction and information on cessation of both e-cigarettes and nicotine products. The franework needs to be reviewed and the intervention co-produced and piloted with key stakeholders and young people, to establish where best to place it within the curriculum and to evaluate its effectiveness at informing young people about e-cigarette use.

## Supplementary Information

**Additional file 1.**

## Data Availability

The dataset and transcripts are not publicly available due to confidentiality and ethical reasons but could be made available from the corresponding author on reasonable request.

## Abbreviations

TPB: Theory of Planned Behaviour
PHA: Public Health Agency
DOH: Department of Health
UK: United Kingdom
ASH: Action on Smoking and Health
NICE: National Institute for Clinical Excellence
EA: Education Authority
UREC: Ulster University Research Ethics Committee
FSM: Free School Meals
TACT: Target, Action, Context and Time
SN: Subjective Norm
PBC: Perceived Behavioural Control
RAG: Research Advisory Group
UAG: User Advisory Group
SPSS: Statistical Package for the Social Sciences
TDF: Theoretical Domains Framework
APEASE: Affordability, Practicability, Effectiveness Acceptability, Side-effects/ safety, and Equity
BCTT: Behaviour Change Techniques Taxonomy
BCW: Behaviour Change Wheel
BCT: Behaviour Change Techniques
NISRA: Northern Ireland Research and Statistics Agency
GCSE: General Certificate of Secondary Education
