# Supplementary material for: Employing the theory of planned behaviour to design an e-cigarette education resource for use in secondary schools
Source: BMC Public Health. 2022 Feb 11;22:276. doi: 10.1186/s12889-022-12674-3 (PMC8832682; doi:10.1186/s12889-022-12674-3)
Supplement: Supplementary file 1 — Additional file 1. [file 12889_2022_12674_MOESM1_ESM.docx]

**Focus group questions**

**Topic 1 - Marketing**

Had you heard of e-cigarettes before today?

Where have you heard about e-cigarettes?

Where have you seen e-cigarettes advertised?

**Topic 2 - Beliefs underlying attitudes.**

What do you believe are the advantages of using e-cigarettes?

What do you believe are the disadvantage of using e-cigarettes?

Is there anything else you associate with your own views about using e-cigarettes?

**Topic 3 – Beliefs underlying subjective norm.**

Who do you think are most likely to use e-cigarettes?

Who do you think are least likely to use e-cigarettes?

Are there any people or groups who would approve of you using an e-cigarette?

Are there any people or groups who would disapprove of you using an e-cigarette?

**Topic 4 – Beliefs underlying control.**

What factors or circumstances would enable you to use e-cigarettes?

What factors or circumstances would make it difficult or impossible for you to use e-cigarettes?

Are there any other issues that come to mind when you think about use e-cigarettes?

**Topic 5 - Outcome expectancy**

What outcomes/consequences would you expect when you use e-cigarettes?

**Topic 6 - Smoking cessation**

Do you think e-cigarettes should be used to help people to stop smoking?

Have you ever used e-cigarettes to quit smoking?

- If so, did they help with this?

Do you think, having used e-cigarettes that people may try ordinary cigarettes?

**Participants were thanked for their participation and asked to contact the researcher by the email provided on the information sheet, if they have any further queries.**
